# Rupture of a giant abdominal aortic aneurysm

**DOI:** 10.1186/1749-8090-8-S1-P11

**Published:** 2013-09-11

**Authors:** Dragan Piljić, Mustafa Tabaković, Almir Kusturica, Alen Hajdarević, Tomislav Klokočovnik

**Affiliations:** 1 Department of Cardiovascular Surgery, University Clinical Center Tuzla, Tuzla, Bosnia and Herzegovina; 2 Department of Cardiovascular Surgery, Ljubljana University Medical Centre, Llubjana, Slovenia

## 

We admitted a 76-year old male person with a ruptured abdominal aortic aneurysm. He was immediately brought to the emergency room (ER). The blood pressure was 80/40 mmHg, HTC less than 0.17. We decided to perform an emergent CT angiography wich verified referral diagnosis. The ruptured AAA was 12 cm in diameter. An emergent life saving operation had to be done so the patient was brought to the operation theatre (OT). The approach to the abdomen was a classic median laparotomy. After we had opened the abdomen, we found a huge retroperitoneal haematoma that pushed beside the intra-abdominal organs rising almost to the edge of the rectal fascia. The blood pressure suddenly dropped down to 45/25 mm Hg for the next several minutes. A continuous infusion of norepinephrine was administered (60 mcg/min) and we also administered several doses of pure adrenalin (3 mg) and pure norepinephrine (1 mg). We pulled out the intestines from the abdominal cavity and clamped the aorta just under the renal arteries. The blood pressure immediately rose to 80 mm Hg. Both common iliac arteries were clamped, too. During the surgery the patient was anuric. Autologous blood transfusion helped by cell saver was administered and additionally he got several doses of blood, blood derivates (fresh frozen plasma, cryoprecipitate, platelets) and other intravenous solutions at the amount of approximately eight litres. We replaced the ruptured AAA using 20 mm PTFE vascular graft. After surgery the patient was transferred to the intensive care unit (ICU) where the blood pressure rose and diuresis was established.

First postoperative day the patient was woken up without neurological deficits. Second postoperative day the patient was able to expel the stool and he was transferred to the department of cardiovascular surgery. Tenth day after surgery he was discharged home. Six weeks after surgery we performed a control CT angiography that showed normal founding on the abdominal iliac and leg vessels.

